# Lactate dehydrogenase-to-albumin ratio as a novel prognostic biomarker for 28-day mortality in thoracic trauma: a single-center retrospective cohort study

**DOI:** 10.55730/1300-0144.6358

**Published:** 2026-04-06

**Authors:** Özgür Ömer YILDIZ, Şükrü YORULMAZ, Eray ÇINAR, Hüseyin MUTLU, Ramiz YAZICI, Ayşenur GÜR, Medine AKKAN ÖZ, Bensu BULUT, Mustafa Sırrı KOTANOĞLU, Hakan GÜNER

**Affiliations:** 1 Department of Thoracic Surgery, Ankara Yenimahalle Education and Research Hospital, Ankara, Turkiye; 2 Department of Emergency Medicine, Ankara Health Practice and Research Center, University of Health Sciences, Ankara, Turkiye; 3 Department of Thoracic Surgery, Ankara Bilkent City Hospital, Ankara, Turkiye; 4 Department of Emergency Medicine, Faculty of Medicine, Aksaray University, Aksaray, Turkiye; 5 Department of Emergency Medicine, Kanuni Sultan Süleyman Education and Research Hospital, University of Health Sciences, İstanbul, Turkiye; 6 Department of Emergency Medicine, Etimesgut Şehit Sait Ertürk State Hospital, Ankara, Turkiye; 7 Department of Emergency Medicine, Gülhane Education and Research Hospital, University of Health Sciences, Ankara, Turkiye; 8 Department of Anesthesia, Ankara Health Practice and Research Center, University of Health Sciences, Ankara, Turkiye; 9 Department of Emergency Medicine, Ankara Etlik City Hospital, Ankara, Turkiye

**Keywords:** Thoracic trauma, LDAR, mortality prediction, biomarker, emergency medicine

## Abstract

**Background/aim:**

Thoracic trauma accounts for approximately 25% of all trauma-related deaths and remains a significant public health challenge. Early identification of high-risk patients is essential for improving clinical outcomes. The present study evaluates the prognostic value of the lactate dehydrogenase-to-albumin ratio (LDAR) in predicting 28-day mortality in patients with thoracic trauma.

**Materials and methods:**

This retrospective observational study was conducted at a Level 1 trauma center between September 2022 and February 2025, and included adult patients presenting with thoracic trauma who underwent thoracic computed tomography. Demographic characteristics, clinical parameters, laboratory values, and 28-day mortality were analyzed. Receiver operating characteristic (ROC) curve analysis was performed to assess the discriminatory power of LDAR and other biomarkers, while multivariate logistic regression was used to identify independent predictors of mortality.

**Results:**

A total of 493 patients were included in the study, 46 of whom (9.3%) died within 28 days. White blood cell count, lactate dehydrogenase, lactate, lactate/albumin ratio, LDAR, and Shock Index were statistically higher in the nonsurvivor group, and albumin, pH, and HCO_3_ levels were lower (p < 0.001). Furthermore, LDAR was significantly higher in the nonsurvivor group when compared to the survivors (13.51 vs. 7.48, p < 0.001). ROC analysis demonstrated that LDAR had excellent discriminatory power for predicting mortality (AUC = 0.854; 95% CI: 0.795–0.914), with an optimal cutoff value of 11.56 yielding 71.7% sensitivity and 91.5% specificity.

**Conclusion:**

LDAR is a reliable and independent prognostic biomarker for predicting in-hospital mortality in patients with thoracic trauma. As a ratio derived from routine laboratory parameters without additional cost, LDAR offers a practical tool for rapid risk stratification and triage optimization in emergency settings. Prospective multicenter studies are warranted to validate these findings.

## 1. Introduction

Trauma is one of the leading causes of death worldwide, particularly among the young and middle-aged, and continues to be a significant public health problem [1]. Thoracic traumas account for approximately 25% of all trauma-related deaths, and is associated with high morbidity [2,3]. Thoracic traumas can be caused by traffic accidents, falls from heights, penetrating injuries, and occupational accidents, and can cause life threatening pathologies such as pneumothorax, hemothorax, and major vascular and cardiac injuries [3]. Timely diagnosis and appropriate interventions can drastically reduce mortality rates [3,4]. The management of patients with thoracic traumas in the Emergency Department (ED), the early identification of high-risk patients, and appropriate triage decisions are of critical importance in improving patient outcomes. While imaging techniques have improved and numerous clinical decision making tools are now available, trauma centers continue their search for reliable, applicable, and effective prognostic indicators [4].

Lactate dehydrogenase (LDH) is an important enzyme that catalyzes the conversion of pyruvate to lactate during glycolysis and has been regarded as an indicator of tissue and organ hypoperfusion [5]. Elevated serum LDH levels have been shown to be associated with poor prognosis in such acute diseases as severe acute pancreatitis, rhabdomyolysis, myocardial infarction, pneumonia, and severe COVID-19 infection [6,7]. Additionally, in cells that have entered a hypoxic-ischemic state, LDH levels start increasing in minutes [8,9]. Serum albumin, on the other hand, is a protein that is synthesized in the liver and has antioxidant and antiinflammatory characteristics, and plays a critical role in regulating plasma osmotic pressure [10–12], while albumin possesses strong antioxidant and antiinflammatory properties, and as a negative acute phase reactant, decreases in conditions such as trauma and sepsis [13,14]. Hypoalbuminemia is a common finding seen in critical patients and is associated with poor prognosis [15].

In recent years, lactate dehydrogenase-to-albumin ratio (LDAR) has been studied as a novel prognostic indicator in various diseases. The ratio combines LDH (a rapid-reacting indicator) and albumin (a slow-reacting indicator) as a potentially useful biomarker for organ failure [16]. LDAR has been observed to be closely associated with prognosis in sepsis, organ failure, tumor, stroke and infectious diseases [17]. Additionally, a positive correlation has been identified between LDAR and both disease severity and mortality risk in patients with severe multitraumas and hemorrhagic shock [18]. However, there have been no studies to date investigating the prognostic value of LDAR in patients with thoracic trauma.

The present study investigates the prognostic value of LDAR in predicting in-hospital mortality in patients with thoracic trauma who presented to a Level 1 trauma center.

## 2. Materials and methods

### 2.1. Study design and participants

**Figure 1:**
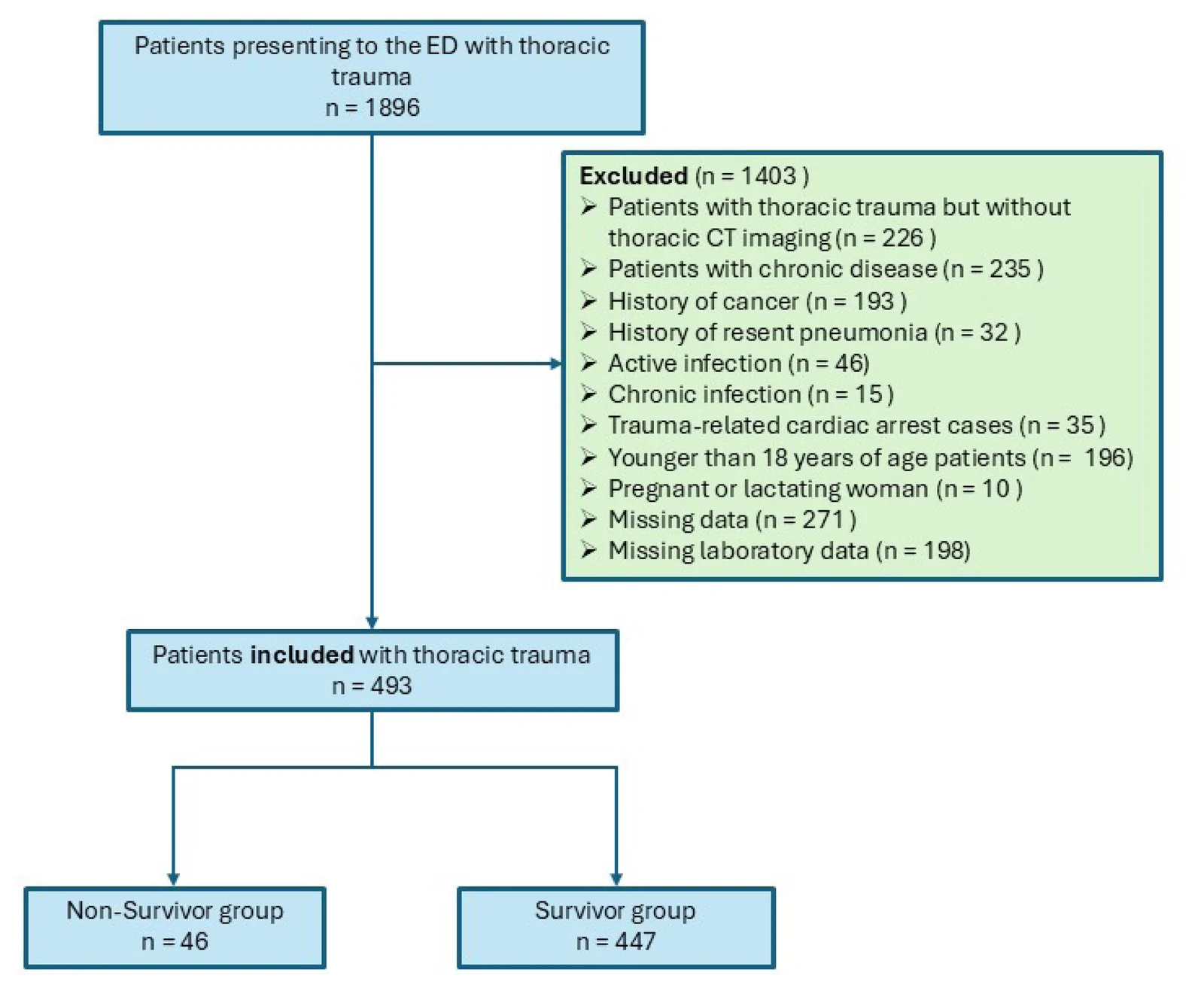
Patient flow chart.

This retrospective observational study was conducted in the Etlik City Hospital Emergency Department between September 28, 2022 and February 1, 2025. The study protocol was approved by the Etlik City Hospital Ethics Committee (No: AEŞH-BADEK-2025-0615 and Date: 26.03.2025), and was conducted in accordance with the principles of the Helsinki Declaration. Owing to the retrospective design of the study, the requirement for informed consent was waived. The hospital in which the study was conducted is a Level 1 trauma center that receives approximately 10,000 trauma patients per month. Included in the study were patients aged ≥18 who presented to our ED with thoracic trauma following traffic accidents, falls from heights, penetrating injuries, and occupational accidents, and who had accessible thoracic computerized tomography (CT) records. Patients were excluded if they had incomplete data; were aged <18 years; were pregnant or lactating; had missing routine blood sample data; had no thoracic trauma, or trauma but no thorax CT imaging records; had known chronic diseases; had a history of cancer; had recent pneumonia; had active or chronic infection; presented to the ED with trauma-related cardiac arrest; or had missing laboratory data. The patient flow of the study is presented in Figure 1.

### 2.2. Study protocol and data collection

Patient data were obtained from the hospital digital data system and patient files. Demographic data such as age, sex, and time of accident were recorded. Various parameters such as vital signs, demographic characteristics, Glasgow Coma Score (GCS), physical examination findings, placement of injury, and complete blood count; as well as creatinine, urea, alanine transaminase (ALT), aspartate aminotransferase (AST), lactate dehydrogenase (LDH), creatinine kinase (CK), and albumin, blood gas and thorax CT results were recorded. Trauma severity was assessed from GCS, Shock Index, and Trauma Severity scores.

Thoracic CT images of the patients were evaluated by experienced radiology specialists and the responsible author B.B. (10 years of ED experience), and classified into six groups: hemothorax, pneumothorax, great artery injury, cardiac injury, lung contusion, and rib fracture. When the evaluations of the author and specialists differed for an individual case, the images were reviewed by another author (H.M., with over 18 years of ED experience) and the final decision was made accordingly. Patients were considered to have great artery injury if they had intimal flap and/or intraluminal thrombus in the great arteries, aortic contour irregularities or caliber changes, and contrast extravasation [19]. Furthermore, patients were considered to have cardiac injury in the presence of ruptures of the free wall of the heart, valve damage, pneumopericardium, cardiac tamponade, or pericardial effusion [20].

The patients were further categorized based on their need for surgical intervention (open thoracotomy or tube thoracostomy). Lactate (mmol/L), LDH (U/L), and albumin (g/L) levels were analyzed from serum plasma samples at the time of presentation to the ED and recorded. LDAR was calculated by dividing serum LDH levels by albumin levels, and the lactate/albumin ratio (LAR) was calculated by dividing lactate levels by albumin levels. Serum albumin levels were measured in the laboratory using the bromocresol green method, while LDH activity was based on the conversion of lactate to pyruvate catalyzed by the LDH enzyme. No changes were made to the analytical methods applied during the data collection or study periods.

Patients were categorized into two groups based on their 28-day survival status as survivors or nonsurvivors.

### 2.3. Statistical analysis

All obtained data were checked for outliers, data integrity, and missing observations prior to the statistical analysis. The distribution of continuous variables was evaluated using the Kolmogorov–Smirnov test. Descriptive statistics were reported as mean ± standard deviation for variables with normal distribution; as median (minimum-maximum) for those without normal distribution; and as numbers and percentages for categorical variables. Inter-group comparisons were performed using Student t-test or a one-way analysis of variance (ANOVA) for continuous variables with normal distribution, and Mann–Whitney U or Kruskal–Wallis tests for variables without normal distribution. Categorical variables were compared using the chi-square (χ^2^) test.

The discriminatory performance of mortality-related potential indicators were assessed using with a receiver operating characteristic (ROC) curve analysis, and the AUC (area under curve), 95% confidence interval (CI) and p-value were reported for each variable. Optimum cut-off points were determined based on the Youden index (J = Se+Sp-1), and the sensitivity, specificity, positive predictive value (PPV), and negative predictive value (NPV) were calculated. Logistic regression analysis was used to determine mortality-related factors, for which each variable was first applied to the model. Univariate logistic regression results were obtained, and variables with p < 0.20 found to be clinically significant were included in the multivariate model. Compatibility with the model was assessed with a Hosmer–Lemeshow compatibility test. Logistic regression results were reported as B coefficient, odds ratio (OR), 95% CI and p-value. A two-tailed p <0.05 was considered statistically significant in all analysis. IBM SPSS Statistics, version 25.0 (IBM Corp., Armonk, NY, USA) was used for the statistical analysis.

## 3. Results

Included in the study were 493 patients who met the study criteria. The median age of the patients was 44 years (interquartile range: 28–61) and 81.9% were male. Males accounted for 60.9% of the nonsurvivor group and 84.1% of the survivor group, and a statistically significant difference between groups was noted in terms of sex (p < 0.001). Of the total, 29.8% of the patients presented to the ED due to traffic accidents, 32.5% due to falls from height, 20.3% due to penetrating injuries, and 17.4% due to other trauma mechanisms. Of the patients with thoracic trauma, 84.1% had rib fractures, 48.3% had pneumothorax, 15.8% had hemothorax, 7.5% had major vascular injury, and 1.4% had cardiac injury. Vascular injuries (23.9% vs. 5.8%) and cardiac injuries (4.3% vs. +1.1) were significantly more common in the nonsurvivor group, indicating a statistically significant relationship between thoracic pathologies and mortality (p <0.001). The demographic and clinical characteristics of the patients are presented in Table 1.

Systolic blood pressure (89 (75–104.5)) and diastolic pressure (72 (59–86)) were significantly lower in the nonsurvivor group (p < 0.001); pulse rates in the nonsurvivor and survivor groups were 119.5 (112–127), 112 (94–125), respectively; and trauma severity scores for the nonsurvivor group and survivor group were 20 (12–25) and 16 (13–19), respectively, and were statistically much higher in the nonsurvivor group (p < 0.001). A comparison of the mortality and hemodynamic parameters in patients with thoracic trauma is presented in Table 2. While WBC, LDH, lactate, LAR, LDAR, and Shock Index were statistically higher in the nonsurvivor group; albumin, pH, and HCO_3_ levels were lower (p < 0.001). The relationship between mortality and laboratory parameters in patients with thoracic trauma is also presented in Table 2.

Statistically significant parameters were included in the regression model. Variables that were found to be significant in the univariate logistic regression analysis were included in the multivariate logistic regression model. Multicollinearity between independent variables was assessed using the variance inflation factor (VIF), producing VIF values of 1.202 for LDH, 1.058 for lactate, and 1.172 for LDAR. In all VIF values <2, no significant multicollinearity was found between the variables. In the multivariate logistic regression analysis, male sex, Trauma Severity Score, LDAR, Shock Index, lactate, and base deficiency were found to be independent predictors of 28-day mortality (p <0.05) (Table 3).

**Figure 2:**
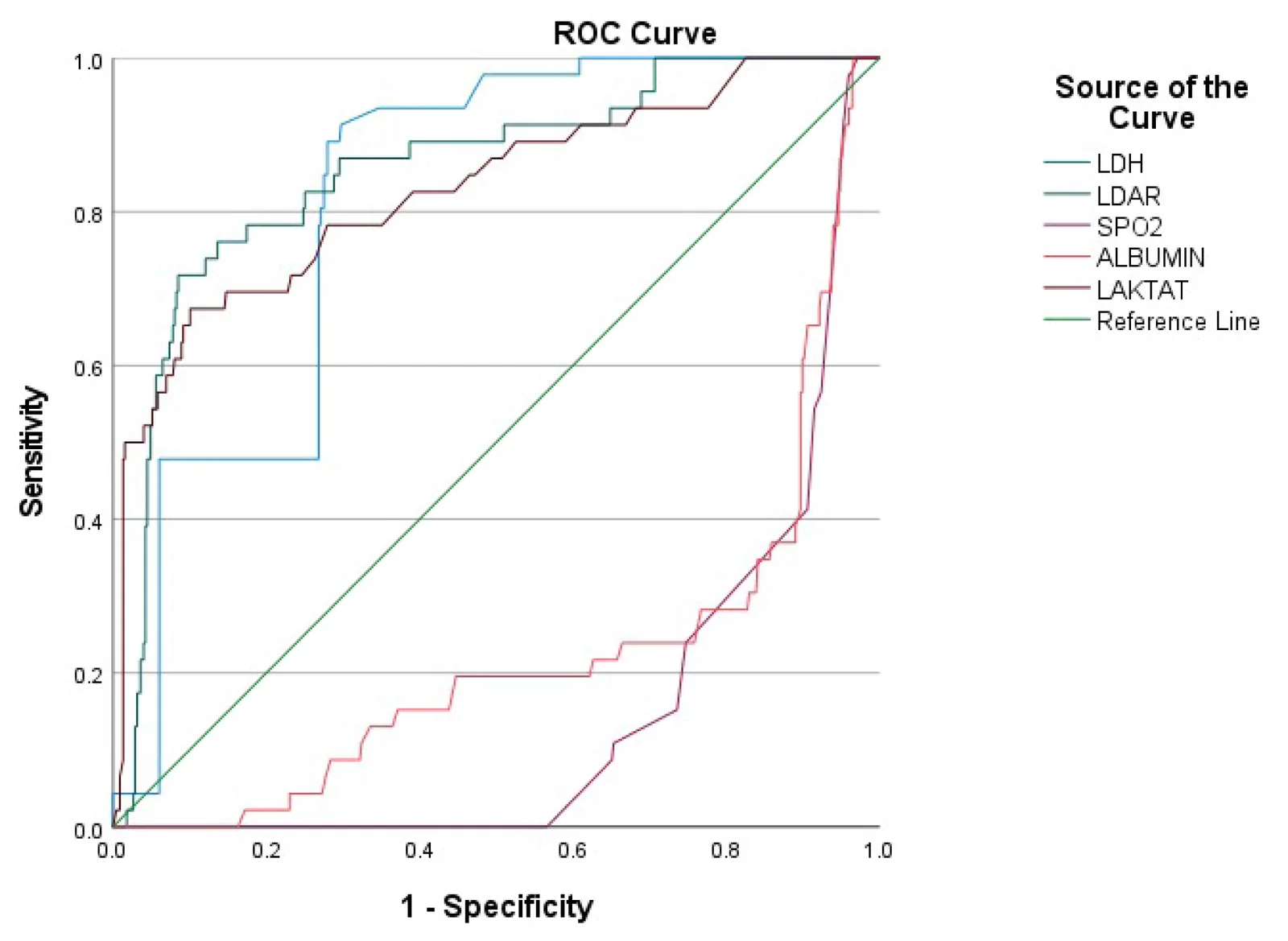
ROC analysis diagram.

The results of a ROC analysis to determine the predictive power for mortality in patients with thoracic trauma revealed the parameter with the highest discriminatory power in predicting 28-day mortality to be LDAR (AUC = 0.854; CI%.795–0.914; p < 0.001) and when optimal cut-off value for LDAR was ≥11.6, sensitivity was 71.7% and specificity was 91.5% (Youden = 0.632). The AUC values of the other parameters were; 0.851 for LAR, 0.845 for SpO_2_, 0.828 for lactate and 0.818 for LDH (Table 4, Figure 2).

## 4. Discussion

Thoracic trauma is a serious clinical condition characterized by high morbidity and mortality rates, and accounts for a significant proportion of ED visits. Timely diagnosis and appropriate interventions can significantly reduce mortality rates. In patients with blunt thoracic trauma, advanced age, three or more rib fractures, and cardiopulmonary disease have been identified as risk factors for increased mortality [21]. Despite the improvements in imaging techniques and the development of various clinical decision making tools, trauma centers are still searching for reliable, applicable, and effective prognostic indicators [4,5]. The aim of the present study was to identify the risk factors for 28-day mortality in patients with thoracic trauma in a Level 1 trauma center. To this end, we investigated the ability of LDAR to predict 28-day mortality and its comparative performance with other inflammatory markers in patients with thoracic trauma. To the best of our knowledge, this is the first study to date analyzing the prognostic value of LDAR in patients with thoracic trauma. In our assessment of the effectiveness of LDAR in predicting in-hospital mortality in patients with thoracic trauma, we found the ratio to be significantly associated with mortality. LDAR values were significantly higher among the patients who died (13.51) when compared to those who survived (7.63) (p < 0.001). The AUC value calculated in the ROC analysis was 0.854, which points to a clinically acceptable discriminatory power. We also noted that advanced age, Trauma Severity Score, Shock Index, lactate, and base deficiency were independent predictors of 28-day mortality.

LDH is an intracellular enzyme found in every cell, and is well-known as an indicator of cellular damage [22]. When cellular damage occurs, LDH leaks out of the cell and this can be measured as an increase in plasma levels. Legrand et al. reported LDH activity to be a reliable indicator of the number of dead cells in a culture environment [23]. In a cell entering a hypoxic/ischemic state, LDH levels start to increase in minutes [9], and LDH is thus associated with poor prognosis and 28-day mortality in the presence of sepsis, acute exacerbation of COPD, pneumonia, ischemic stroke, and acute renal failure, among others [6, 24, 25, 26]. The anaerobic glycolysis that occurs during the hypoxic and ischemic processes following trauma causes a significant increase in LDH activation [9]. We also found serum that LDH levels had an AUC value of 0.815 in predicting mortality, suggesting that in cases of thoracic trauma, pulmonary contusion, myocardial damage, and musculoskeletal injuries can lead to increased LDH levels, making it a reliabler biomarker of tissue damage and prognosis.

Serum albumin is a protein that is synthesized in the liver and has antiinflammatory and antioxidant properties, and is also known to play a critical role in various physiological processes, such as the regulation of plasma osmotic pressure [10]. Goldwasser and Feldman reported a significant relationship between serum albumin concentration and mortality risk [27]. This was supported by Yap et al., who also reported a strong correlation between serum albumin levels and mortality risk [28], while Cao et al. found albumin levels to be associated with both short- and long-term mortality in sepsis patients [29]. Corroborating these studies, albumin levels in the present study were significantly lower in the mortality-group (32.6 g/L vs. 41.4 g/L, p < 0.001) and ROC analysis revealed a high discriminatory power in predicting mortality (AUC = 0.779). Hypoalbuminemia may serve as an indicator of both acute phase response and the catabolic processes associated with trauma.

Although LDH and albumin individually have prognostic value in various clinical conditions, using them independently can be limiting as both can be affected by many factors [30,31]. For this reason, LDAR—the ratio LDH to albumin—stands out as a more comprehensive and accurate prognostic indicator for the assessment of such factors as organ failure, chronic disease, inflammation, and nutritional status together. The pathophysiologic mechanisms responsible for the prognostic value of LDAR are multifaceted. Experimental studies have revealed that LDH plays a critical role in the pathogenic inflammation that occurs in the Akt/HIF1α/LDH signal pathway during ischemia-reperfusion damage. Chowdhury et al. reported that elevated LDH increases tissue acidity, triggering the formation of abnormal invasive-like protrusions in neutrophils and inducing transcellular migration via endothelial cells, thereby increasing vascular leakage. The pathogenic inflammatory state of ischemia/reperfusion injury is associated with increased neutrophil transcellular migration and vascular leakage [30, 32]. Conditions such as pulmonary contusion, pneumothorax and hemothorax that occur following thoracic trauma cause tissue hypoxia and ischemia, increasing the release of LDH. Albumin, on the other hand, has strong antioxidant and antiinflammatory properties and, as a negative acute-phase reactant, decreases during the systemic inflammatory response following trauma [13,14]. Yan et al. reported a significant association between high LDAR values and the risk of stroke-related pneumonia [33]. Jeon et al., on the other hand, reported an association between high LDAR levels and increased mortality in intensive care unit (ICU) patients [34]. In their retrospective cohort study conducted on critical acute respiratory distress syndrome (ARDS) patients, they reported that LDAR was predictive of 30-day mortality [24]. Guan et al. reported LDAR to be associated with all-cause mortality during ICU stay in sepsis patients [36]. In a study of the prognostic value of LDAR in critical patients with acute renal disease, Deng et al. reported high LDAR levels to be independently associated with mortality [37]. The AUC value of 0.878 in the present study indicated the strong discriminatory performance of LDAR in predicting mortality, with a cut-off value of 8.03 yielding 87% sensitivity and 84.8% specificity.

Lactate levels are commonly used when assessing prognosis in trauma patients as a reliable indicator of tissue hypoperfusion and anaerobic metabolism. Lactate levels increase due to reduced microcirculation and are affected by low arterial oxygen levels, leading to increased lactic acid production through anaerobic glycolysis. Çınar et al., in their study of patients with thoracic trauma, found that each 1-unit increase in lactate levels was associated with a 1.9-fold increase in mortality [38]. In the present study, lactate levels were significantly higher in the mortality group (4.39 mmol/L vs. 1.53 mmol/L, p < 0.001) and the multivariate analysis revealed that each 1-unit increase in lactate levels doubled the mortality risk (aOR = 1.96). The AUC of 0.828 indicated strong discriminatory power in the ROC analysis. These findings are congruent with those of Yazıcı et al. showing a strong relationship between lactate levels and mortality in patients with isolated thoracic trauma [39].

Hemodynamic instability is one of the key factors affecting mortality in trauma patients. Shock Index is obtained by dividing pulse rate by systolic blood pressure, and is a practical scoring system based on physiology that is a more reliable predictor of mortality than either hypotension or tachycardia by themselves [40]. In their study of trauma patients, Cannon et al. demonstrated the effectiveness of the Shock Index in predicting mortality [40]. Furthermore, in a comprehensive study of 16,269 patients conducted by McNab et al., a high Shock Index was associated with an increased mortality rate [41]. Olaussen et al., on the other hand, reported that Shock Index values in excess of 0.9 were predictive of massive hemorrhage, and that treatment should be planned accordingly [42]. In our study, a cut-off value of 1.16 yielded high sensitivity (97.8%) and moderate specificity (44.1%), supporting the use of the Shock Index as a screening tool. Furthermore, our multivariate analysis showed that each 1-unit increase in the Shock Index was associated with an approximate 9.3-fold increase in mortality risk (OR = 9.27), highlighting the critical prognostic significance of hemodynamic instability in patients with thoracic trauma.

Our study has several strengths. Firstly, to the best of our knowledge it is the first study to date investigating the role of LDAR in predicting mortality in patients with thoracic trauma. LDAR is an easily applicable biomarker that has strong discriminatory power in predicting in-hospital mortality in patients with thoracic trauma. Additionally, the fact that it can be easily calculated using routine biochemistry tests with no additional costs are further significant advantages. Among the limitations, the study’s retrospective design makes it difficult to establish a causal relationship, and some confounding factors may not have been controlled. Furthermore, the single-center study design limits the generalizability of the results, and pre-hospital factors that can affect mortality, such as pre-hospital response times or transport conditions, were not evaluated. Finally, no consecutive LDAR measurements were made and the prognostic value of dynamic changes was not evaluated. In clinical practice, calculating LDAR in patients with thoracic trauma can contribute to the early identification of high-risk patients and the optimization of treatment strategies. Such findings may be confirmed through prospective and multicenter studies in the future.

## 5. Conclusion

Our study reveals LDAR to be a strong and independent biomarker that can predict in-hospital mortality. LDH and albumin are routine laboratory tests and can be obtained without any additional costs, supporting the use of LDAR as a practical tool for rapid risk assessment in the emergency department and in environments with limited resources. Our results suggest that LDAR can be utilized as an assistive parameter in the triage and planning of treatment in patients with thoracic trauma. Future large-scale multicenter and prospective studies involving various trauma populations are required in order to determine optimal cut-off values for integration into clinical decision-making algorithms.

## Figures and Tables

**Table 1:** Comparison of demographic, clinical, and intervention variables related to mortality in patients with thoracic trauma.

	Total (n = 493)	Nonsurvivor (n = 46)	Survivor (n = 447)	p Value
**Age** Median [IQR]	44 (28–61)	42.50 (27.50–61.25)	44 (28–61)	0.938**
**Sex**				
Male	404 (81.9)	28 (60.9)	376 (84.1)	<0.001*
Female	89 (18.1)	18 (39.1)	71 (15.9)	
**Trauma Mechanism**				
Traffic accident	147 (29.8)	21 (45.7)	126 (28.2)	0.084
Fall from height	160 (32.5)	10 (21.7)	150 (33.6)	
Injury from sharp objects	100 (20.3)	9 (19.6)	91 (20.4)	
Other	86 (17.4)	6 (13.0)	80 (17.9)	
**GCS Score**				
3–8	21 (4.3)	8 (17.4)	13 (2.9)	<0.001*
9–12	19 (3.9)	8 (17.4)	11 (2.5)	
13–15	453 (91.9)	30 (65.2)	423 (94.6)	
**Thoracic Pathology**				
Rib fracture	415 (84.1)	44 (95.6)	371 (83.0)	<0.001*
Lung contusion	254 (51.5)	43 (93.5)	211 (47.2)	<0.001*
Pneumothorax	238 (48.3)	21 (45.7)	217 (48.5)	0.708
Hemothorax	78 (15.8)	4 (8.7)	74 (16.6)	0.164
Vascular injury	37 (7.5)	26 (56.5)	11 (2.5)	<0.001*
Cardiac injury	7 (1.4)	7 (15.2)	0 (0.0)	<0.001*
**Accompanying Injury**				
No	144 (29.2)	6 (13.0)	138 (30.9)	0.011*
Yes	349 (70.8)	40 (87.0)	309 (69.1)	
**RBC Transfusion**				
No	459 (93.1)	35 (76.1)	424 (94.9)	<0.001*
Yes	34 (6.9)	11 (23.9)	23 (5.1)	
**Intubation**				
Not performed	449 (91.1)	14 (30.4)	435 (97.3)	<0.001*
Performed	44 (8.9)	32 (69.6)	12 (2.7)	
**Tube Thoracostomy**				
Not performed	275 (55.8)	11 (23.9)	264 (59.1)	<0.001*
Performed	218 (44.2)	35 (76.1)	183 (40.9)	
**Emergency Surgery**				
Not performed	286 (58.0)	17 (37.0)	269 (60.2)	0.002*
Performed	207 (42.0)	29 (63.0)	178 (39.8)	

* Ki-square test,

** Mann-Whitney U test, p < 0.05 statistically significant

**IQR:** Interquartile Range; **GCS:** Glasgow Coma Score; **RBC**: Red Blood Cell

**Table 2:** Comparison of mortality and associated hemodynamic parameters and laboratory indicators in patients with thoracic trauma.

	Total (n = 493)	Nonsurvivor (n = 46)	Survivor (n = 447)	p Value
**Systolic** Median (IQR)	89 (75–104.50)	79.50 (70.75–87.00)	91 (75–106)	<0.001*
**Diastolic** Median (IQR)	72 (59–86)	57.50 (53.75–62.50)	74 (61–88)	<0.001*
**Pulse** Median (IQR)	113 (96–126)	119.50 (112.0–127.0)	112 (94–125)	0.002*
**SpO_2_** Median (IQR)	89 (84–91)	83 (80–84.25)	89 (84–91)	<0.001*
**Trauma Severity Score** Median (IQR)	16 (13–20)	20 (15–25)	16 (13–19)	<0.001*
**WBC** Median (IQR)	12.48 (9.38–16.17)	14.12 (9.73–17.83)	12.40 (9.31–16.13)	0.040*
**Hemoglobin** Median (IQR)	14.10 (12.80–15.30)	13.65 (11.77–15.05)	14.10 (12.80–15.40)	0.101
**Platelet** Median (IQR)	225 (172.50–281.00)	236.50 (135.50–326.00)	225 (178–279)	0.946
**Neutrophil** Median (IQR)	9.18 (6.32–13.07)	9.97 (7.24–15.09)	9.16 (6.20–12.85)	0.055
**Lymphocyte** Median (IQR)	1.63 (1.19–2.55)	2.46 (1.21–4.02)	1.61 (1.19–2.46)	0.023*
**Creatinine** Median (IQR)	0.86 (0.73–1.01)	0.97 (0.81–1.28)	0.86 (0.73–0.99)	0.001*
**Urea** Median (IQR)	31.40 (24.50–40.55)	32.35 (23.52–41.72)	31.40 (24.50–40.50)	0.923
**LDH** Median (IQR)	296 (287–390)	379 (368.75–472.00)	294 (286–383)	<0.001*
**Albumin** Median (IQR)	41.10 (37.30–43.90)	32.60 (29.20–38.55)	41.40 (38.40–44.10)	<0.001*
**ALT** Median (IQR)	22 (15–38)	29 (19–73.50)	21 (15–36)	0.005*
**AST** Median (IQR)	29 (21–52)	37 (22.75–128.00)	29 (21–49)	0.008*
**pH** Median (IQR)	7.35 (7.35–7.40)	7.33 (7.27–7.37)	7.35 (7.35–7.41)	<0.001*
**HCO_3_** Median (IQR)	21.30 (17.30–24.70)	17.50 (12.40–20.10)	21.70 (19.30–24.90)	<0.001*
**Lactate** Median (IQR)	1.60 (1.23–2.14)	4.39 (1.82–7.38)	1.53 (1.23–1.84)	<0.001*
**Base deficiency** Median (IQR)	–2.20 [(–5.40)–(1.10)]	–5.40 [(–9.25)–(–3.47)]	–1.80 [(–5.40)–(1.30)]	<0.001*
**NLR** (Neutrophil/Lymphocyte)	5.23 (3.05–9.60)	4.41 (2.60–9.96)	5.34 (3.12–9.58)	0.593
**LAR** (Lactate/Albumin)	0.03 (0.03–0.05)	0.14 (0.05–0.22)	0.03 (0.02–0.05)	<0.001*
**LDAR** (LDH/Albumin)	7.63 (6.80–9.70)	13.51 (10.18–14.94)	7.48 (6.73–9.20)	<0.001*
**Shock index** (Pulse/Systolic)	1.25 (1.03–1.53)	1.52 (1.29–1.68)	1.21 (1.02–1.48)	<0.001*

* Mann Whitney U Test, p < 0.05 statistical significance

**IQR:** Interquartile Range; **SpO_2_:** Peripheral Oxygen Saturation; **WBC:** White Blood Cell; **LDH:** Lactate Dehydrogenase; **ALT:** Alanine Transaminase; **AST:** Aspartate Aminotransferase.

**Table 3:** Mortality-related factors in patients with thoracic trauma: Uni- and multivariate regression analysis.

Variable	Category/Reference	B coefficient	Univariate OR	95%CI	p value	Multivariate OR	95%CI	p Value
**Gender Sex**	Male	1.225	3.404	1.788–6.483	<0.001*	5.150	1.828–14.510	0.002*
**GCS (3**–**8)**	13–15	2.161	0.115	0.044–0.300	<0.001*			
**Accompanying Trauma**	No	1.091	2.977	1.233–7.187	0.015*			
**Trauma Severity Score**		0.092	1.096	1.053–1.142	<0.001*	1.098	1.031–1.170	0.004*
**LDAR**		0.092	1.096	1.053–1.142	<0.001*	0.907	0.852–0.966	0.002*
**Shock Index**		0.136	0.873	0.815–0.935	<0.001*	0.932	0.891–0.976	0.003*
**ISS**	Mild to moderate trauma	0.712	2.038	1.028–4.040	0.041*			
**Systolic**	Hypotension	−1.634	0.195	0.089–0.428	<0.001*			
**Diastolic**	Hypotension	−1.817	0.162	0.086–0.308	<0.001*			
**Respiratory physiology**	Low risk	2.877	17.769	5.950–53.068	<0.001*			
**LDH**		0.208	1.232	1.047–1.450	0.012*			
**Albumin**		−0.093	0.911	0.881–0.943	<0.001*			
**HCO** **_3_**		−0.171	0.843	0.790–0.899	<0.001*			
**Lactate**		0.628	1.875	1.600–2.197	<0.001*	1.959	1.571–2.444	<0.001*
**Base deficiency**		−0.252	0.777	0.712–0.849	<0.001*	0.770	0.679–0.872	<0.001*
**RBC transfusion**	Not performed	1.757	5.794	2.611–12.854	<0.001*			
**Intubation**	Nor performed	4.417	82.857	35.396–193.958	<0.001*			
**Tube thoracostomy**	Not performed	1.524	4.590	2.272–9.273	<0.001*			
**Emergency surgery**	Not performed	0.947	2.578	1.376–4.830	0.003*			

* p <0.05 statistically significant

**CI:** Confidence Interval; **OR:** Odds Ratio; **GSC:** Glasgow Coma Score; **LDAR:** LDH/Albumin ratio; **ISS:** Injury Severity Score; **LDH:** Lactate Dehydrogenase; **RBC:** Red Blood Cell

**Table 4:** ROC analysis of biomarkers and clinical scores predicting mortality in patients with thoracic trauma.

Variables	AUC	p value	95% CI	Cut-off	Sensitivity	Specificity	Youden Index	PPV	NPV
**SpO** **_2_**	0.854	<0.001*	0.814–0.895	87.50	91.3	65.1	0.564	21.2	98.6
**Albumin**	0.779	<0.001*	0.706–0.853	36.55	71.7	82.8	0.545	30.0	96.6
**Base deficiency**	0.753	<0.001*	0.682–0.824	–1.75	93.5	49.7	0.431	16.0	98.6
**Trauma Severity Score**	0.687	<0.001*	0.593–0.782	19.50	63.0	76.1	0.391	21.3	95.2
**Shock Index**	0.725	<0.001*	0.666–0.784	1.16	97.8	44.1	0.419	15.2	99.4
**LDH**	0.815	<0.001*	0.767–0.863	329.00	89.1	72.0	0.612	24.6	98.4
**Lactate**	0.828	<0.001*	0.756–0.899	2.75	67.4	89.9	0.573	40.7	96.4
**LAR**	0.851	<0.001*	0.786–0.916	0.07	67.4	87.5	0.549	32.9	96.2
**LDAR**	0.854	<0.001*	0.795–0.914	11.56	71.7	91.5	0.632	46.4	96.9

* p < 0.05 statistically significant

**AUC:** Area Under the Curve; **CI:** Confidence Interval; **PPV:** Positive Predictive Value; **NPV:** Negative Predictive Value; **SpO2:** Peripheral Oxygen Saturation; **LDH:** Lactate Dehydrogenase; **LAR:** Lactate/Albumin ratio; **LDAR:** LDH/Albumin ratio.
